# Safety and feasibility study of using polyphosphate (PolyP) in alveolar cleft repair: a pilot study

**DOI:** 10.1186/s40814-021-00939-4

**Published:** 2021-11-08

**Authors:** Salem A. Alkaabi, Diandra Sabrina Natsir Kalla, Ghamdan A. Alsabri, Abul Fauzi, Nova Jansen, Andi Tajrin, Rifaat Nurrahma, Werner Müller, Heinz C. Schröder, Wang Xiaohong, Tymour Forouzanfar, Marco N. Helder, Muhammad Ruslin

**Affiliations:** 1 Department of Oral and Maxillofacial Surgery/Oral Pathology, Amsterdam University Medical Centers and Academic Centre for Dentistry Amsterdam (ACTA), Vrije Universiteit Amsterdam, Amsterdam Movement Sciences, Amsterdam, The Netherlands; 2 Department of Oral and Maxillofacial Surgery, Fujairah Hospital, Ministry of Health, Fujairah, United Arab Emirates; 3 Department of Biochemistry, Faculty of Medicine, Hasanuddin University, Makassar, Indonesia; 4 Department of Oral and Maxillofacial Surgery, Faculty of Dentistry, Hasanuddin University, Makassar, Indonesia; 5 Institute for Physiological Chemistry, University Medical Center, University Mainz, Mainz, Germany; 6 Institute NanotecMARIN GmbH, Mainz, Germany; 7 Department of Prosthodontic, Faculty of Dentistry, Hasanuddin University, Makassar, Indonesia

**Keywords:** Polyphosphate, Alveolar bone grafting, Bone regeneration, Regenerative medicine

## Abstract

**Background:**

Bone grafting is an important surgical procedure to reconstruct alveolar bone defects in patients with cleft lip and palate. Polyphosphate (PolyP) is a physiological polymer present in the blood, primarily in platelets. PolyP plays a role as a phosphate source in bone calcium phosphate deposition. Moreover, the cleavage of high-energy bonds to release phosphates provides local energy necessary for regenerative processes. In this study, polyP is complexed with calcium to form Calcium polyP microparticles (Ca-polyP MPs), which were shown to have osteoinductive properties in preclinical studies. The aim of this study was to evaluate the feasibility, safety, and osteoinductivity of Ca-polyP MPs, alone or in combination with BCP, in a first-in-human clinical trial.

**Methods:**

This single-blinded, parallel, prospective clinical pilot study enrolled eight adolescent patients (mean age 18.1: range 13–34 years) with residual alveolar bone cleft. Randomization in two groups (four receiving Ca-polyP MPs only, four a combination of Ca-polyP MPs and biphasic calcium phosphate (BCP)) was performed. Patient follow-up was 6 months. Outcome parameters included safety parameters and close monitoring of possible adverse effects using radiographic imaging, regular blood tests, and physical examinations. Osteoinductivity evaluation using histomorphometric analysis of biopsies was not possible due to COVID restrictions.

**Results:**

Due to surgical and feasibility reasons, eventually, only 2 patients received Ca-polyP MPs, and the others the combination graft. All patients were assessed up to day 90. Four out of eight were able to continue with the final assessment day (day 180). Three out of eight were unable to reach the hospital due to COVID-19 restrictions. One patient decided not to continue with the study.

None of the patients showed any allergic reactions or any remarkable local or systematic side effects. Radiographically, patients receiving Ca-polyP MPs only were scored grade IV Bergland scale, while patients who got the BCP/Ca-polyP MPs combination had scores ranging from I to III.

**Conclusions:**

Our results indicate that Ca-polyP MPs and the BCP/Ca-polyP MPs combination appear to be safe graft materials; however, in the current setting, Ca-polyP MPs alone may not be a sufficiently stable defect-filling scaffold to be used in alveolar cleft repair.

**Trial registration:**

Indonesian Trial Registry under number INA-EW74C1N by the ethical committee of Faculty of Medicine, Hasanuddin University, Makassar, Indonesia with code number 1063/UN4.6.4.5.31/PP36/2019.

## Introduction

### Background

Cleft lip and palate (CLP) are common anomalies in the craniofacial region and are considered the second most common congenital deformity after the clubfoot [1]. An alveolar cleft is seen in 75% of the CLP patients [2, 3]. Alveolar bone grafting (ABG) is an essential functional and esthetic procedure to reconstruct the bony defect in the maxilla as well as the nasal floor [4]. ABG not only plays an important role to facilitate teeth eruption, but also to fill the bony defect by closing the oronasal fistula that routinely occurs in alveolar cleft patients.

The alveolar bone grafting can be performed using autogenous bone, allograft bone, or bone substitutes. Autogenous bone graft is still considered as the gold standard for any grafting procedure [5]; nevertheless, numerous studies are employing various bone substitutes or allografts to overcome the risks and complications that could raise from harvesting bone at the donor site [6–8]. Risks such as gait disturbance, hematoma, donor site morbidity, and other concerns that are associated with the growth (through harvesting from the rib or the iliac crest) could be avoided if a good allograft or bone substitute material would be available [9].

Polyphosphate (polyP) is a molecule that is naturally present in platelets in the blood stream. Müller and his colleagues have been able to structure a new graft material by precipitation of polyP with calcium, thus forming Ca-polyP microparticles (Ca-polyP MPs) [10–12]. The Ca-polyP MPs were proven to have bone osteoinductive characteristics in preclinical studies [12–14]. It has been shown that the Ca-polyP MPs can accumulate and concentrate at the site of the new bone formation. PolyP polymer elicits both the anabolic signals and the fuel due to energy-rich phosphate anhydrides linkages as well as the metabolic process in the cells. Such signals could accelerate the cell growth and differentiation [15].

On the other hand, Biphasic calcium phosphate (BCP) is another type of graft that contains a phosphate molecule mixed with hydroxyapatite (HA) in different ratios. Ambivalent outcomes have been reported to BCP as graft material; some stated that BCP has osteoconductive characteristics [16, 17], while others concluded that it also can be osteoinductive in nature [18, 19].

### Objective

This first-in-human study aims to evaluate the safety, feasibility, and osteoinductivity of Ca-polyP MPs, alone or in combination with BCP, as a graft material in alveolar cleft patients.

## Material and methods

### Ethics

This single-blinded, prospective clinical trial, a pilot study was approved by the ethical committee of Faculty of Medicine, Hasanuddin University, Makassar, Indonesia, with code number 1063/UN4.6.4.5.31/PP36/2019. It was registered in the Indonesian Trial Registry under number INA-EW74C1N. The study protocol complies with the principles of the Helsinki declaration. Patients and legal guardians of the patients signed an informed consent.

No special ethical approval was required for this study.

### Patients and randomization

This study enrolled eight patients with residual alveolar bone cleft. The inclusion criteria were non-syndromic, nonsmoker, age of ≥ 13, no history of previous grafting procedure(s), and ASA1 regarding anesthetic risks. The exclusion criteria were systemic diseases, syndromic patients, localized infection, active influenza, obvious malnutrition, or patient under any active medical treatment. Randomly using closed envelopes, four out of eight patients were selected to receive the Ca-polyP MPs alone, while the other four patients were to receive a mixture of Ca-polyP MPs and BCP as a graft material. However (see the “Results” section), eventually, two patients only received Ca-polyP MPs alone, while six received the mixture. The surgeon and the patients were revealed to the graft type; nevertheless, the assessor was kept completely blinded from the patient grouping. The time schedule of the surgical procedure and follow-up moments is presented in Table 1.

**Table 1:** Treatment time schedule

	Consent form	Panorama	CBCT or CT	Physical examination	CBC	Thermometer	Biopsy
**Preoperatively**	**✓**	**✓**	**✓**	**✓**	**✓**	**✓**	
**Operative day**				**✓**		**✓**	
**Post-op day 1**		**✓**		**✓**	**✓**	**✓**	
**Post-op day 8**		**✓**	**✓**	**✓**	**✓**	**✓**	
**Post-op day 14**				**✓**		**✓**	
**Post-op day 30**				**✓**	**✓**	**✓**	
**Post-op day 90**		**✓**		**✓**		**✓**	
**Post-op day 180**		**✓**	**✓**	**✓**	**✓**	**✓**	**✓**

*OPG* orthopantomogram, *CT* computed tomography, *CBCT* cone beam CT

### Sample size

Since this is a first-in-human trial, the number of patients was kept low in order to minimize the risk of the graft exposure in case of any adverse effect. The current trial sample was limited to only 2 × 4 patients, with the primary goal to gain a first insight on the feasibility and safety of the treatment with polyP.

### Randomization and treatment allocation

After written informed consent, randomization was performed with regard to the treatment group, but all patients were aware of the fact that their treatment comprised Ca-polyP MPs.

### Blinding

The radiologist remained blind to the treatment when evaluating the data.

### Data collection

Doctors, nurses, and the rest of the research team were provided with a list of rules and responsibilities. The doctors and nurses collected the data according to the assessment Table 1. All research team members received training on how to collect data at all study visits. Each patient has been followed up to 6 months. Patient confidentiality was protected by the data manager.

### Polyp and BCP preparation

**Fig. 1:**
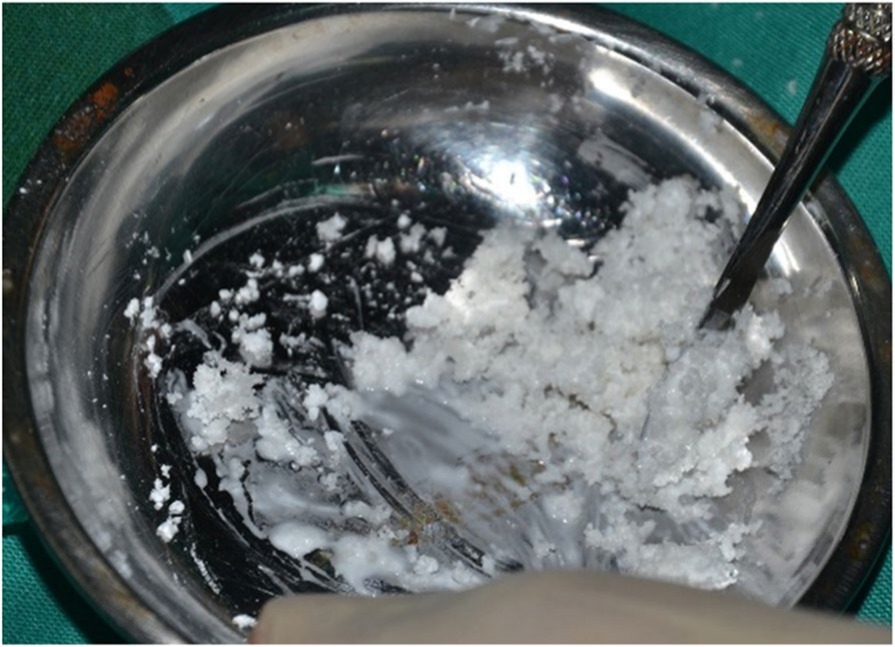
Ca-polyP MP + BCP mixed with normal saline

PolyP graft comes in a form of Ca-polyP MPs powder produced by NanotecMARIN GmbH (Mainz, Germany), while the BCP consists of a mixture of 60% hydroxyapatite and 40% of beta-tricalcium phosphate (Straumann Bone Ceramic, Villeret, Switzerland). Under sterile conditions, either Ca-polyP MPs or a mixture of Ca-polyP MPs and BCP was prepared using normal saline at a ratio of 1 g:1.5 ml and 1 g:2 g:3–5 ml respectively. The components were mixed until a homogenous mixture was obtained (Fig. 1).

### Surgical procedure

**Fig. 2:**
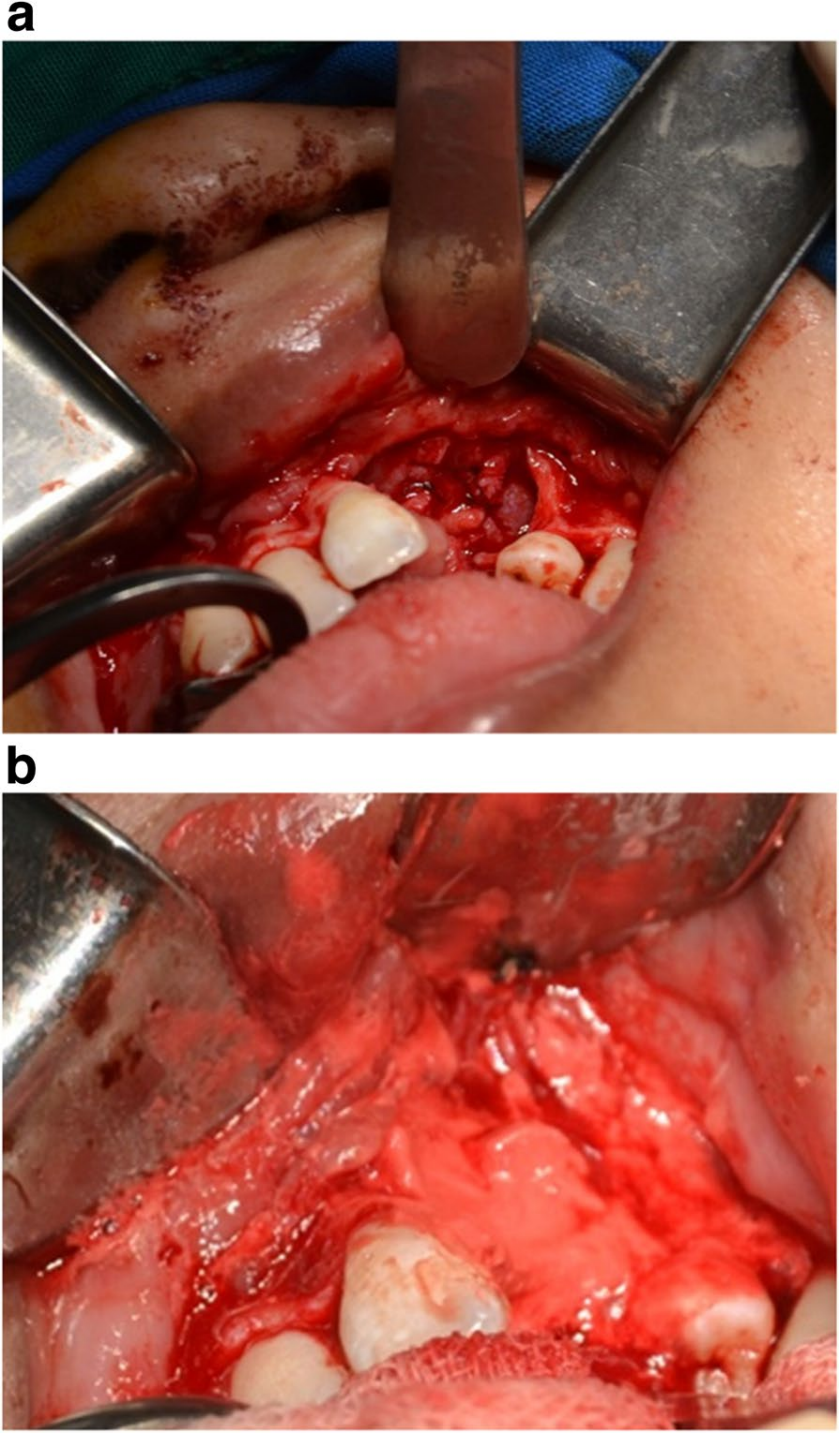
**a** Nasal floor reconstruction and exposing the bony edges. **b** Ca-polyP MPs graft placed in the defect

Under general anesthesia and full aseptic conditions, the oral cavity was rinsed with 0.1% chlorhexidine gluconate solution. A local anesthesia infiltration using lidocaine with epinephrine 1:100,000 was given. Full mucoperiosteal flap was reflected from the first molar to the central incisor on the contralateral side of the defect. The tissue was dissected carefully to separate the oral mucosa from the nasal layer. A palatal mucoperiosteal flap was reflected from either side of the cleft followed by elevation of the palatal tissues. The nasal mucosa was cranially elevated and sutured cranially to repair the oro-nasal fistula (Fig. 2a). A Ca-polyP MPs preparation or the Ca-polyP MPs and BCP mixture was applied into the alveolar cleft defect (Fig. 2b). Tension-free closure was realized in all wounds.

#### Postoperative care

Oral hygiene instructions were given to all patients including mouth rinsing with 0.12% Chlorhexidine. Antibiotics (amoxicillin/clavulanic acid) and pain killers were prescribed for 7 days according to the standard of care. During hospital stay, follow-up examinations of all patients were meticulously performed to report any adverse reaction to the grafting materials locally or systemically. After patient discharge, all patients followed an assessment timetable.

### Orthopantomogram (OPG)

#### Bergland scale

OPGs were taken one day preoperatively (X-Mind Pano D+ Satelec- Digital panoramic with teleradiography - Satelec), and then subsequently after 8, 30, 90, and 180 days. The OPGs were used to assess the vertical graft formation employing the Bergland scale, which is the gold standard used to evaluate the integrity and height of the alveolar bone graft [20]. The Bergland scale is classified into four grades: grade I, bone height is almost a normal height; grade II, a bone height at least 75% of the interalveolar septum; grade III, the bone height is less than 75%; and grade IV, no evidence of bone integration [21].

### CT scan

The CT scans (Siemens SOMATOM Definition Flash CT Scanner) were performed preoperatively and at postoperative days 8 and 180. The data were processed by OsiriX (Pixmeo, Switzerland), an open-source Digital Imaging and Communications in Medicine (DICOM).

## Results

All patients were able to comply with the study requirements up to assessment day 90. Unfortunately, four out of eight patients were unable to continue with the final assessment (day 180). One patient decided not to continue with the study, while the other three patients were unable to approach the hospital due to the COVID-19 lockdown at their towns/villages (Table 2).

**Table 2:** Demographic and assessment data

	Pt.1	Pt.2	Pt.3	Pt.4	Pt.5	Pt.6	Pt.7	Pt.8
**Gender**	F	F	M	F	F	F	F	F
**Age**	18	13/14	13	15	13	15	24	34
**Affected side**	Left	Left	Bilateral	Left	Right	Left	Right	Left
**Graft type**	Ca-polyP MPs	Ca-polyP MPs	Ca-polyP MPs + BCP	Ca-polyP MPs + BCP	Ca-polyP MPs + BCP	Ca-polyP MPs + BCP	Ca-polyP MPs + BCP	Ca-polyP MPs + BCP
**Assessment day 30**	Completed	Completed	Completed	Completed	Completed	Completed	Completed	Completed
**Assessment day 90**	Completed	Completed	Completed	Completed	Completed	Completed	Completed	Completed
**Assessment day 180**	Missed follow-up, COVID-19 lockdown	Completed	Completed	Completed	Missed follow-up, COVID-19 lockdown	Missed follow-up, COVID-19 lockdown	Drop-out	Completed

*Pt.* patient, *F* female, *M* male, *Ca-polyP* calcium polyphosphate microparticles, *BCP* biphasic calcium phosphate

All eight patients underwent bone grafting surgery by the same surgeon. There were no reported postoperative complications, local or systematic, in both study groups. All patients were in close follow-up from day 1 until they were discharged from the hospital (day 3). Thereafter, the patients were followed up according to Table 2. Although not included in the initial trial design, all patients were contacted with video or telephone calls up for a 1-year follow-up. No adverse events were reported, and all patients reported that they were content with the treatment.

### Feasibility

Two different application modes of Ca-polyP-MP should have been tested in a randomized manner, but as a consequence of the difficulty to handle Ca-polyP microparticles when not complexed with BCP, we had to abandon the randomization of graft type and applied the BCP-polyP graft type only. Thus, feasibility appeared valid for the combination graft, but not (in the current setting) for the application of Ca-polyP MPs only.

### Safety

#### Adverse events

The main goal of this study was to evaluate the safety of the Ca-polyP MPs, alone or in combination with BCP, in terms of adverse events (local or systemic) using clinical assessment, radiographic, and laboratory investigations (a.o. white blood cells, neutrophil, lymphocyte, and if needed C-reactive protein) (Table 3). All patients were kept hospitalized postoperatively for 72 h to maintain close follow-up. In the case of SAE concerning severe toxicity or infection associated with the graft site, the trial would be terminated immediately.

**Table 3:** Safety assessments

	Pt.1	Pt.2	Pt.3	Pt.4	Pt.5	Pt.6	Pt.7	Pt.8
**Graft type**	Ca-polyP MPs	Ca-polyP MPs	Ca-polyP MPs + BCP	Ca-polyP MPs + BCP	Ca-polyP MPs + BCP	Ca-polyP MPs + BCP	Ca-polyP MPs + BCP	Ca-polyP MPs + BCP
**Pain**	Mild	Mild	Minimum pain/pressure	Mild	Mild	Minimum pain/pressure	Mild	Moderate
**Fever**	No	No	No	No	No	No	No	No
**Allergic reaction**	ND	ND	ND	ND	ND	ND	ND	ND
**Remarkable local inflammation/infection**	No	No	No	No	No	No	No	No
**Systematic adverse effect**	ND	ND	ND	ND	ND	ND	ND	ND
**Lab tests**	Within normal limits	Within normal limits	Within normal limits	Within normal limits	Within normal limits	Within normal limits	Within normal limits	Within normal limits

*Ca-polyP MPs* calcium polyphosphate microparticles, *BCP* biphasic calcium phosphate, *ND* nothing detected

### Osteoinductivity

Since the acquirement of biopsies was not possible due to COVID-19 restrictions, this aspect could not be evaluated as planned [22].

### Radiographic evaluation

#### Orthopantomogram

The Bergland scale was used in this study to investigate the result of the secondary bone grafts in alveolar defects. This scale is considered the gold standard to assess the post alveolar graft height of the interdental septum. Although OPG is more susceptible to distortions, it was chosen because it is more patient-friendly when compared to the other intra-oral x-rays, especially when taken postoperatively.

In the Ca-polyP MPs group (patients 1 and 2), bone levels were not suitable to be analyzed with the Bergland scale, and we decided to score them as grade IV bone level at all assessment days (Table 4). One of these patients could not attend the last follow-up session (day 180). In the Ca-polyP MPs-BCP group, the bone level ranged from grade I to III in assessment days 1, 8, and 90. Only three patients could be assessed at day 180, and all of them had grade III bone level (Table 4).

**Table 4:** Bergland scores based on OPGs

Bergland scale	Ca-Polyp MPs graft		Ca-Polyp MPs + BCP					
	Pt.1	Pt.2	Pt.3	Pt.4	Pt.5	Pt.6	Pt.7	Pt.8
**Day 1**	IV	IV	I	I	II	I	I	I
**Day 8**	IV	IV	I	I	II	I	I	I
**Day 90**	IV	IV	III	III	III	III	II	III
**Day 180**	ND	IV	III	III	ND	ND	ND	III

*ND* no data

### CT scan evaluation

As indicated above, the bone levels in the Ca-polyP MPs group could not be analyzed with the Bergland scale. The material had a ground glass appearance (scattered light radiopaque). Since no bone level could be identified we classified them as grade IV at both day 8 and day 180. In the Ca-polyP MPs-BCP group, the CT scans showed a differential bone level from grade I to grade III per patient (Table 5). For the last three patients who could be scanned at day 180, bone levels were found to be consistent with those determined with the OPG, i.e., grade III Bergland scale.

**Table 5:** Bergland scores based on CTs scan

Bergland scale	Ca-polyP MPs graft		Ca-polyP MPs + BCP					
	Pt.1	Pt.2	Pt.3	Pt.4	Pt.5	Pt.6	Pt.7	Pt.8
**Day 8**	IV	IV	I	II	I	III	I	II
**Day 180**	Missed follow-up, COVID-19 lockdown	IV	III	III	Missed follow-up, COVID-19 lockdown	Missed follow-up, COVID-19 lockdown	Drop-out	III

*a-polyP MPs* calcium polyphosphate microparticles, *BCP* biphasic calcium phosphate

### Complications

There were no complications reported intra- and/or postoperatively in both study groups.

## Discussion

In the current trial, we found that Ca-polyP MPs appear to be a safe material: no unusual adverse reactions were reported, such as infection, severe pain, swelling, allergic reaction, or any other local or systemic adverse effects. With regard to the feasibility, the microparticles probably may need a stable graft material such as BCP for appropriate alveolar reconstruction.

The optimum age for the alveolar bone grafting is considered to be between 9 and 11 years old [20, 23]. Since we did not want to enroll children in a safety study with this novel material in clinical practice, we chose to only include older adolescent and adult patients, being capable themselves to make sound decisions. We performed this study in Indonesia because non-operated patients in this age group are difficult to find in Europe.

In the Ca-polyP MPs group, the main challenge was in the handling and application of the material in the alveolar defect. The characteristics of the Ca-polyP MPs can be determined by Pi: Ca+2 molar ratio. In our trial, we used a paste-like mixture formed by mixing fine Ca-polyP MPs graft with normal saline as described in the materials and methods. However, the resulting Ca-polyP MPs graft material was easily lost from the surgical sites once it got saturated with blood, which made maintaining a space-occupying scaffold within the alveolar defect virtually impossible. We therefore had to conclude that the physical characteristics of the Ca-polyP MPs used as a stand-alone scaffold material were insufficient and unfeasible. As a consequence, we had to reduce the Ca-polyP MPs only group to 2 instead of 4 patients as planned originally in the study protocol. Retrospectively, the reason that the microparticles were previously shown to be effective in bone formation in preclinical studies may be due to the location used: it was implanted in a subcutaneous pocket instead of a not well contained, large void such as the alveolar cleft [24, 25].

Combining the Ca-polyP MPs with BCP considerably improved the consistency, ease of handling, stability of the graft, and clinical outcome. BCP and calcium phosphates in general have been used as a graft material several times in craniofacial surgery before. For example, Levitt et al. already used calcium phosphate in 1969 for this purpose, and calcium phosphates were subsequently used in dental implant, alveolar ridge augmentation, periodontal treatment, and other maxillofacial surgeries. Biphasic calcium phosphate (BCP) has been proven to be biocompatible and exhibit osteoconductive as well as osteoinductive characteristics in bony defects reconstruction [16, 17, 19]. Calcium phosphate was also recently applied in alveolar cleft surgery [26]. Based on our results, we recommend that to achieve the feasibility of applying bioactive Ca-polyP MP, it should be combined with a stable carrier such as BCP or bioresorbable polymers to ensure proper reconstructive activity. Likely, special attention should be paid to sequestration of the polyP on or within the carrier, of which we could not be sure in the current study.

Our study was limited by several aspects, the most severe being the COVID-19 pandemic allowing only 4 patients to be evaluated after 180 days of follow-up and thereby resulting in a rather short postoperative follow-up period. Another limitation was the rather radiolucent characteristic of the Ca-polyP MPs, which hampered visualization of the graft in radiographic images considerably and making an evaluation with the Bergland scale virtually impossible. We also tried the Chelsea scale [27], which analyzes the bone position in relation to the adjacent teeth on the grafting site radiographically. However, this did not result in other outcomes as the Bergland scale, so we omitted these results. We can therefore not be completely sure whether defect filling was sufficient and if some initial bone regeneration events occurred, but at least no solid bone formation was demonstrated after 3 months, and also not in the one patient evaluated after 6 months. Last but not least, it may be that the choice to include only adolescent and adult people in our study and to exclude prepuberal children may have affected the efficacy of the treatment. Bone formation activity usually has its highest peak during puberty, and our post-puberal patient population may therefore have more restricted bone formation capacity per se. In addition, the cleft defects in our patients were mostly rather large, thus reducing the likeliness of effective bone regeneration as well.

To our knowledge, this study is the first clinical trial to investigate the safety and feasibility of polyP, either as Ca-polyP MPs alone or in combination with BCP in humans. A histological examination of the bone at six months was not performed due to the COVID 19 restrictions in Indonesia, which hampered osteoinductivity assessment considerably. We could now only evaluate this aspect based on the radiographic results.

Despite this limitation, since we have now performed video/phone calls at 1 year postoperative, and all patients did report that they had no adverse events and that they were content with the treatment, we can deduce that the treatment with polyP-containing grafts may be safe and in combination with BCP appears to be feasible for alveolar cleft repair. Nevertheless, new studies with a larger group of patients, biopsy evaluations, and suitable polyP formulations encompassing appropriate carriers such as BCPs or polymeric scaffold materials are required for sound conclusions about their regenerative capacities. Eruption of the teeth through the site, periodontal and health of the root surface of the adjacent teeth, orthodontic movement of adjacent teeth to the grafted site need to be taken into account as well.

## Conclusions

Despite the small sample group size and some missing data points due to the COVID-19 pandemic, we were able to conclude that Ca-polyP MPs and the Ca-polyP MP/BCP composites appear to be safe graft materials, however, Ca-polyP MPs alone may not be a sufficiently stable defect-filling scaffold to be used in alveolar cleft repair.

## Data Availability

Availability of data and materials: Original data is stored securely within the Hasanuddin University, Makassar, Indonesia. Scored date and output for analyses are available upon request from the study.

## Abbreviations

PolyP: Polyphosphate
Ca-polyP MPs: Calcium polyP microparticles
BCP: biphasic calcium phosphate
CLP: Cleft lip and palate
ABG: Alveolar bone grafting
HA: Hydroxyapatite
